# Design strategy of surface decoration for efficient delivery of nanoparticles by computer simulation

**DOI:** 10.1038/srep26783

**Published:** 2016-05-26

**Authors:** Hong-ming Ding, Yu-qiang Ma

**Affiliations:** 1 Center for Soft Condensed Matter Physics and Interdisciplinary Research, Soochow University, Suzhou 215006, China; 2 National Laboratory of Solid State Microstructures and Department of Physics, Collaborative Innovation Center of Advanced Microstructures, Nanjing University, Nanjing 210093, China

## Abstract

Understanding the role of surface decoration of nanoparticles in protein adsorption and cellular uptake is of great importance in biomedicine. Here, by using dissipative particle dynamics simulations, we take two typical coating polymers (i.e., hydrophilic and zwitterionic polymers) as an example, and systematically investigate their effect on cellular delivery of hydrophobic and charged nanoparticles (in the presence of serum protein). Our results show that though two types of polymers are charge-neutral and can both reduce the protein adsorption, there exist some differences between their ability of protein resistance, especially in the case of positively charged nanoparticles. Besides, it is found that the coating polymers may also greatly decrease the cellular uptake efficiency of nanoparticles. Nevertheless, and importantly, since the zwitterionic polymers may become positively charged under low pH environments, the nanoparticle can attach onto cell membrane more firmly than that coated with hydrophilic polymers, which can further enhance the active targeting of nanoparticles. Finally, we also provide the design maps for surface decoration to achieve efficient cellular delivery. These results can help better understand how to keep the balance between protein resistance and cell targeting, which may give some useful guidelines on optimal design of future nanomaterials in drug delivery.

Nowadays, efficient delivery of nanomaterials and specific biomacromolecules to the interior of targeted cells (e.g., cancer cells) is of great importance in nanomedicine^1^,^2^. Recently, it has been realized that tumor vasculature may have defective architecture with pore sizes ranging from 100 to 780 nm, allowing for extravasation of nanoparticles within this size range^3^. Besides, tumor tissues usually lack effective lymphatic drainage, which prevents efficient removal of nanoparticles from tumors^4^. These phenomena are known as the enhanced permeability and retention (EPR) effect^5^. To better take advantage of the EPR effect, nanoparticles must circulate for a prolonged period in the body. However, there exist plenty of plasma proteins in the blood, and they can adsorb on the nanoparticle surface due to different nonspecific interactions (e.g., hydrophobic and electrostatic interactions)^6^,^7^,^8^, which may initialize the immune response and remove nanoparticles out of the blood^9^,^10^.

In order to avoid the clearance of nanoparticles by phagocytic cells, one effective way is to decorate hydrophilic and non-charged polymers, such as polyethylene glycol (PEG) and polysaccharide, onto the nanoparticle surface^11^,^12^,^13^. Moreover, zwitterionic polymers, containing both cationic and anionic groups, can be also used and are even believed to have some advantages over hydrophilic polymers^14^,^15^. Generally, these stealth coatings can greatly resist the adsorption of serum proteins, which will increase the circulation half-life of nanoparticles. However, it should be noted that these polymers will also affect the subsequent interaction of nanoparticles with cancer cells, and may have some side effects on the cellular uptake^7^,^11^. Therefore, it is necessary and important to keep the balance between protein resistance and cellular uptake. Further, it is also of great importance and urgency to reveal the disparity between these different types of coatings and further clarify their distinct roles in cellular delivery.

Actually, there have been great experimental progresses on these problems in recent years^16^,^17^,^18^,^19^,^20^. For example, Dawson *et al.*^16^ illustrated that the protein corona can screen the targeting molecules on the surface of nanoparticles and induce the loss of their targeting capabilities; Chan *et al.*^17^ found that the coating PEG can reduce the formation of the protein corona, but may also interfere with the binding of the targeting ligand to its corresponding cellular receptor in some cases. Nevertheless, limited by the available experimental technologies, it is still difficult to systematically probe and visualize the whole cellular delivery process under various conditions. Computer simulation, on the contrary, can provide some useful insights into the molecular mechanism of these problems^21^,^22^. And with the advance in computer technology and simulation methods, there have been many computational works on cell-nanoparticle interactions, especially for the role of physicochemical properties of nanoparticles in the cellular uptake^23^,^24^,^25^,^26^,^27^,^28^,^29^. However, to the best of our knowledge, presently there do not exist any simulation studies to well address the above issues.

In this work, we undergo the computational study to systematically investigate the role of surface decoration in the cellular delivery of nanoparticles by using dissipative particle dynamics (DPD) simulations^30^,^31^. The ability of two typical and widely used coatings (i.e., hydrophilic and zwitterionic polymers) to resist protein adsorption on the nanoparticle surface will be firstly examined. Then we will compare their distinct effects on the interactions between nanoparticles and cell membranes. Finally, the optimal design strategy in the presence of antibody for efficient delivery of nanoparticles will be discussed in details.

## Results

### Construction of different components in simulation system

**Figure 1:**
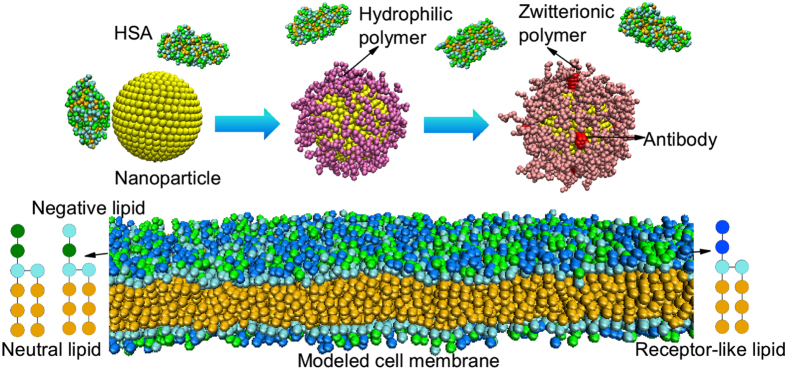
Schematic illustration of the coarse-grained models in the DPD simulations. The bead of hydrophilic charged nanoparticles is yellow while the bead of hydrophobic ones is ochre; the bead of antibody is red; the bead of hydrophilic polymer is mauve while that of zwitterionic polymer is pink. The green bead represents charged head in lipid molecule (the first green bead containing +*e* and the second one containing −*e*) and HSA protein, while lime bead stands for lipid head and hydrophilic part of HSA protein without charges, the orange bead represents lipid tail and hydrophobic part of HSA protein, the blue bead stands for receptor head bead. Water and ion beads are not shown for clarity.

Figure 1 illustrates the coarse-grained models of different components in the simulations. The nanoparticle is fabricated by arranging DPD beads on a fcc lattice with lattice constant *α* = 0.40 *nm* into a desired geometry shape and volume, and all beads comprising a nanoparticle move as a rigid body^32^,^33^. The polymer covalently decorated on the nanoparticle surface is composed of several connected beads (the number of the beads can be varied)^23^,^34^. For hydrophilic polymer (HP), the beads are all hydrophilic and non-charged^23^. For zwitterionic polymer (ZP), the ending two beads are charged, where the last bead carries one negative charge and the penultimate bead carries one positive charge^14^. Since here we just focus on the difference of charge property between these two types of polymers, the remaining beads of zwitterionic polymer are hydrophilic and non-charged, which are the same as that of hydrophilic polymer. Besides, for the sake of simplicity, the antibody here is just treated as a hydrophilic rigid cylinder^35^, and its length is fixed as 2.5 nm.

When modeling the cell membrane, we firstly use the charge-neutral lipid molecules that consist of a headgroup containing four connected hydrophilic beads and two tails with respective three hydrophobic beads to self-assemble into lipid bilayers^24^,^36^, where the first head bead carries a charge of +*e*, the second head bead carries a charge of −*e*, and the remaining two beads are uncharged^37^. Besides, experimental results have shown that there are more anionic molecules (e.g., phosphatidylserine and heparan sulfate proteoglycans) abundant on the surface of cancer cells as compared to normal cells^38^. To mimic the negative charge property of cancer cells, here for the sake of simplicity, we set ten percent of lipid molecules as negatively charged ones in our simulations^39^. And when modeling the negatively charged lipids, non-charged hydrophilic bead is used to replace the first positively charged bead in lipid molecule^39^,^40^. Moreover, we also use a simplified model (i.e., the receptor-like lipid) to simulate the specific transmembrane protein, where the receptor-like lipid has the same conformation of lipid molecule^23^,^41^, but its first two head beads are uncharged and can interact with the antibody bead via soft Lennard-Jones (LJ) potentials^32^.

The protein used in this work is human serum albumin (HSA), which is the most abundant protein in human blood plasma and constitutes about half of the blood serum proteins^42^. Similar to previous coarse-grained simulations and theories^43^,^44^,^45^, here we use a coarse-grained model for the HSA protein, namely, each amino acid is represented by a single bead and the bead type is determined by the amino acid residue^43^,^44^,^45^. The beads are connected by harmonic bonds into a freely jointed linear chain, whose sequence is in line with amino acid sequence of HSA protein (PDB file:1AO6)^46^. Additionally, the monovalent ion and water beads are also introduced into the simulation system, where the ion concentration is about 0.10 M, close to that in physiological conditions.

### Role of surface decoration in protein resistance

Previous experimental and computational studies have shown that nanoparticles (especially the hydrophobic and positively charged ones) without any surface decoration could be quickly covered by different types of proteins when inserted into the blood^6^,^16^,^44^. This may shorten the circulation time and greatly decrease the delivery efficiency. In order to prolong the circulation time, different types of non-specific polymers need to be decorated onto the nanoparticle surface. Here we choose two typical types of polymers, namely hydrophilic polymer and zwitterionic polymer, and check their ability of protein resistance.

**Figure 2:**
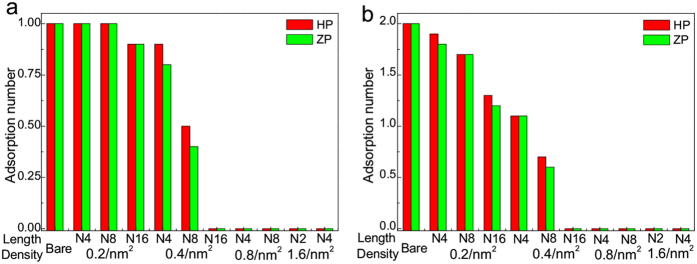
Interaction of HSA protein with hydrophobic nanoparticles. The adsorption number of HSA protein on hydrophobic nanoparticle surface as functions of the property (i.e., polymer length and density) of coating polymers (ten independent simulations are averaged). (**a**) The nanoparticle size is 6 nm; (**b**) the nanoparticle size is 10 nm.

For hydrophobic nanoparticles, in the absence of surface polymers, the HSA protein will adsorb on the nanoparticle surface, with some of its hydrophobic parts interacting with nanoparticles^8^,^44^. When decorating with hydrophilic polymers, since its surface becomes partially hydrophilic, the probability of protein adsorption will decrease. As shown in Fig. 2, with the increase of surface density (*σ*), the adsorption number decreases very quickly. On the contrary, increasing the polymer length (N), especially under the low surface density (e.g., 0.2/*nm*^2^), just has little impact on the protein resistance. In this sense, for hydrophobic nanoparticles, the surface density of its coating polymers should be the most important factor in protein resistance. Besides, it is also found that there exists little difference of adsorption number among the two types of polymers, indicating that the resistance ability is nearly independent on the polymer type. This is because here the hydrophobic interaction is the dominant force for adsorption, while the electrostatic interaction is not very important. Further, we should notice that though the adsorption number depends on the nanoparticle size, the minimal surface density for protein resistance seems to be independent on the size. As shown in Fig. 2a,b, when the surface density is larger than 0.4/*nm*^2^, the adsorption numbers are both greatly reduced no matter what the nanoparticle size and polymer length are. Interestingly, we notice that this phenomenon is consistent with a previous experimental study^47^, namely, there also existed one critical surface density (i.e., *σ* = 0.5/*nm*^2^), at which the proteins can hardly adsorb onto the hydrophobic surface.

For charged nanoparticles, for the sake of simplicity, here we just consider the homogenously charged nanoparticles with no hydrophobic part on their surface, which is beneficial for determining the role of electrostatic interaction in the protein adsorption^44^. In addition, since the HSA protein can hardly adsorb on negatively charged nanoparticles (with no hydrophobic part) due to the repulsively electrostatic interaction^44^, it is not necessary to coat stealth polymers onto their surface for HSA protein resistance. But notice that the surface of negatively charged nanoparticles in real experiments may be semi-hydrophilic or partially hydrophobic, thus the HSA protein could adsorb onto their surface^48^. However, the main driving force for protein adsorption in those cases is hydrophobic interaction (instead of electrostatic interaction)^48^, which is similar to that in the case of hydrophobic nanoparticles. As a result, here we just focus on the case of positively charged nanoparticles.

**Figure 3:**
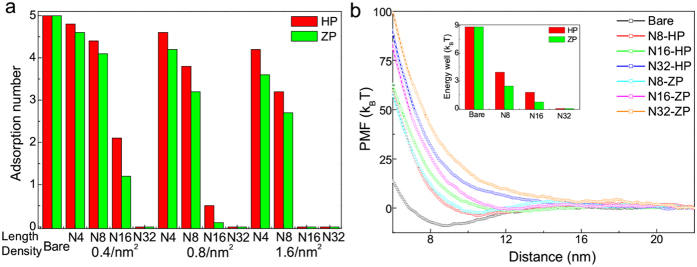
Interaction of HSA protein with positively charged nanoparticles. (**a**) The adsorption number of HSA protein on positively charged nanoparticle surface as functions of the property (i.e., polymer length and density) of coating polymers where the nanoparticle size is 10 nm and the surface charge density is 0.2 *e*/*nm*^2^ (ten independent simulations are averaged). (**b**) Potential of mean force of adsorption as a function of the distance from center of mass of nanoparticle to that of HSA protein under different surface coatings (the polymer density is 0.8/*nm*^2^). The inset shows the depth of energy well (*i.e.*, the minimal value) in the PMF profile (five independent simulations are averaged).

Since the electrostatic interaction is long-ranged, in order to avoid the adsorption of HSA protein (onto positively charged nanoparticles), polymers with longer length are required. As shown in Fig. 3a, the adsorption number just decreases a little when the positively charged nanoparticle is decorated with shorter polymers (e.g., N = 4 or 8), even under high surface density 1.6/*nm*^2^. On the contrary, when decorated with the longer polymers (e.g., N = 16), the nanoparticle could totally resist the adsorption of protein under middle surface density (e.g., 0.8/*nm*^2^). In this sense, here the polymer length should be the most important factor. But we should notice that once the polymer length is long enough, the protein resistance ability will change little with the increase of polymer length. This length-dependent effect is consistent with the experimental result^49^, where the total amount of adsorbed proteins on nanoparticle surface is decreased when coated with longer polymers. Interestingly, their results also showed that there was no obvious difference of protein adsorption between very long polymers-coated nanoparticles with further increase of the polymer length^49^. Besides, here the polymer type also has some impacts, i.e., there are some differences between the hydrophilic and zwitterionic polymers. Considering that the HSA protein is negatively charged and the last bead of zwitterionic polymer carries one negative charge, thus there may exist the charge mismatch of the charged parts between zwitterionic polymers and HSA protein, which induces the weakly repulsive electrostatic interaction between them (Fig. S1). As a result, the adsorption of HSA protein onto zwitterionic polymers-coated nanoparticles will become a bit more difficult. We also calculate the potential of mean force (PMF) profile of single HSA protein interacting with charged nanoparticles under different situations by using steering molecular dynamics (with the pulling velocity of about 0.00167 nm/ns)^50^. As shown in Fig. 3b, the PMF for most nanoparticles (except for the coating polymer length N = 32) decreases with the decrease of distance between the nanoparticle and HSA protein. However, the depth of the energy well in PMF is different. And with the increase of polymer length, the depth becomes smaller and may disappear under the case of very long polymers (see the inset in Fig. 3b). Moreover, we also find that the depth of HP-coating nanoparticles is slightly larger than that of ZP-coating nanoparticles, indicating that the ability of zwitterionic polymer for protein resistance is a bit better, which is in agreement with recent experimental findings^51^. Generally, despite the difference of atomistic structures between the hydrophilic polymer (e.g., PEG) and zwitterionic polymer (e.g., polysulfobetaine)^15^, here we demonstrate that the general difference of charge property between them can also have great impact on their protein resistance ability.

### Role of surface decoration in cellular uptake

As discussed above, the decoration of stealth polymers onto nanoparticle surface can reduce the probability of protein adsorption, which is beneficial for the cellular delivery. However, since the surface property of nanoparticles is changed, it may also alter the subsequent interaction of nanoparticles with cell membranes. In this part, we will investigate how these coating polymers affect the efficiency of cellular uptake.

**Figure 4:**
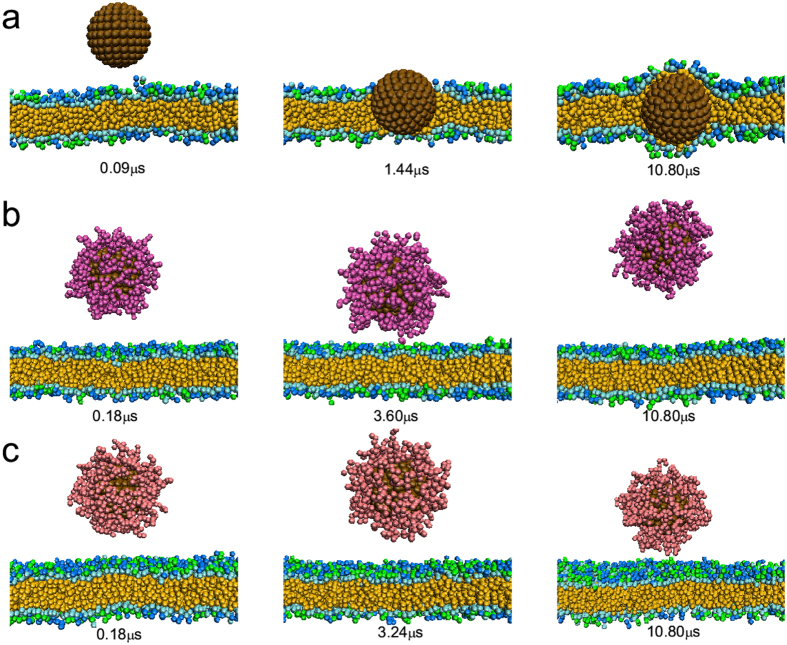
Time sequence of the snapshots of interactions between hydrophobic nanoparticle with different surface coatings and cell membranes where the nanoparticle size is 6 nm. (**a**) Bare nanoparticle; (**b**) nanoparticle decorated with hydrophilic polymers (N = 8, *σ* = 0.8/*nm*^2^); (**c**) nanoparticle decorated with zwitterionic polymers (N = 8, *σ* = 0.8/*nm*^2^).

For hydrophobic nanoparticles, as shown in Fig. 4a, the bare hydrophobic nanoparticle can insert into the membrane interior spontaneously. While decorated with hydrophilic polymers (N = 8, *σ* = 0.8/*nm*^2^, the proteins cannot adsorb onto the nanoparticle surface under this situation), since its surface becomes hydrophilic, the nanoparticle can no longer insert into the interior of lipid bilayer and may even leave far away from the membrane (Fig. 4b). Thus the uptake or penetration efficiency of hydrophobic nanoparticles will become lower. Similarly, in the case of zwitterionic polymers, the insertion of hydrophobic nanoparticle is also blocked. However, there may exist dipole-dipole interaction between zwitterionic polymers and charge-neutral lipid molecules, thus the zwitterionic polymers-coated nanoparticle may weakly adsorb on the membrane surface (Fig. 4c), inducing the little decrease of total energy between nanoparticle (including polymers) and cell membrane (see Table 1).

**Figure 5:**
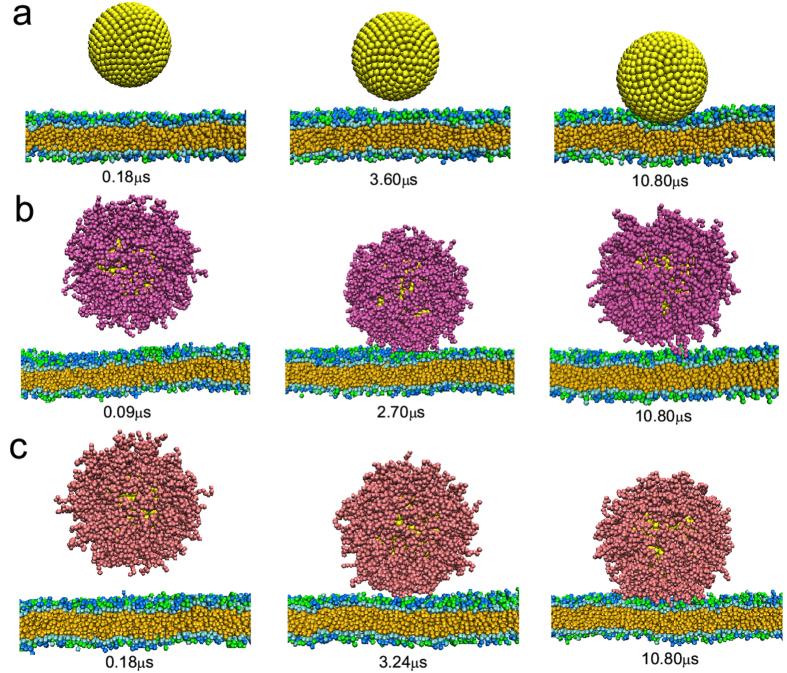
Time sequence of the snapshots of interactions between positively charged nanoparticle with different surface coatings and cell membranes where the nanoparticle size is 10 nm and the surface charge density is 0.2 *e*/*nm*^2^. (**a**) Bare nanoparticle; (**b**) nanoparticle decorated with hydrophilic polymers (N = 16, *σ* = 0.8/*nm*^2^); (**c**) nanoparticle decorated with zwitterionic polymers (N = 16, *σ* = 0.8/*nm*^2^) under low pH (one third of the zwitterionic polymers are ionized and each carries one positive charge).

For positively charged nanoparticles, the presence of coating polymers also seems to have negative effects on the cell-nanoparticle interaction. As shown in Fig. 5a, the electrostatic interaction between the positive nanoparticle and negative membranes can induce the adsorption of the nanoparticle. Nevertheless, when decorated with hydrophilic polymers (N = 16, *σ* = 0.8/*nm*^2^, under this situation nearly no proteins adsorb on the nanoparticle surface), these hydrophilic polymers will “screen” the electrostatic interaction so that the interaction strength becomes much weaker. In this sense, the efficiency of cellular uptake is greatly reduced (Fig. 5b). Such similar phenomenon is also observed in the case of positively charged nanoparticles coated with zwitterionic polymers (see Table 1, *d* and Δ*E* are very close in the two cases). However, some types of zwitterionic polymers are pH-responsive and may carry positive charges at specific pH value^52^,^53^. For example, PCL-b-P(AEP-g-TMA/DMA) shows nearly neutral charge at pH 7.4 (~ blood and normal tissue pH) and becomes positively charged at pH 6.8 (~ tumor tissue pH)^53^. This can enhance the electrostatic interaction between the polymer-coated nanoparticle and negatively charged membranes. As shown in Fig. 5c, there exists obvious deformation of the coating polymers on the lower surface of nanoparticle. Moreover, this also induces the deformation of cell membrane, but the deformation is very small (Fig. S2) because the rigidity of cell membrane is larger than that of polymer-coated nanoparticles^28^. Generally, the final equilibrium distance between the nanoparticle and cell membrane becomes much smaller, indicating that the adsorption of nanoparticle onto cell membrane becomes much stronger. Interestingly, we notice that previous experimental study has found that the uptake of pH-responsive zwitterionic polymer-coated nanoparticles by cancer cells is more efficient than that coated with non-responsive charge-neutral polymers, which leads to a much higher tumor therapy efficiency in the case of zwitterionic polymer-coated nanoparticles^53^. Thus our simulation results here can give a reasonable explanation for the experimental observation. While for negatively charged nanoparticles, since the cell membrane is also negatively charged, they cannot approach onto the cell membrane no matter whether there exist polymeric coatings (see Table 1). This phenomenon is also consistent with the experimental results^54^, where it was found that the cellular uptake efficiency of negatively charged nanoparticles is lower than that of positively charged nanoparticles.

## Discussion

The above results have shown that the decoration of non-specific (hydrophilic or zwitterionic) polymers onto the nanoparticle surface may have Janus faces on the cellular delivery, namely, on one hand, it can resist the adsorption of serum protein, on the other hand, it may decrease the translocation efficiency of nanoparticles through membranes. Though the electrostatic interaction between nanoparticles (and/or zwitterionic polymers) and cell membranes may induce the adsorption of nanoparticles, the adsorption may be not strong enough to initiate the active endocytosis of nanoparticles. In order to enhance the targeting ability, different types of antibodies, along with non-specific polymers, could be decorated on the nanoparticle surface^16^,^17^. Here we will discuss the optimal design strategy in the presence of polymers and antibody to achieve high delivery efficiency. And for the sake of simplicity, each nanoparticle is decorated by six antibody molecules. Since the antibody molecule is hydrophilic and the surface density is very low, the coating of antibody molecules onto nanoparticle surface has very little impact on the HSA adsorption (Fig. S3).

**Figure 6:**
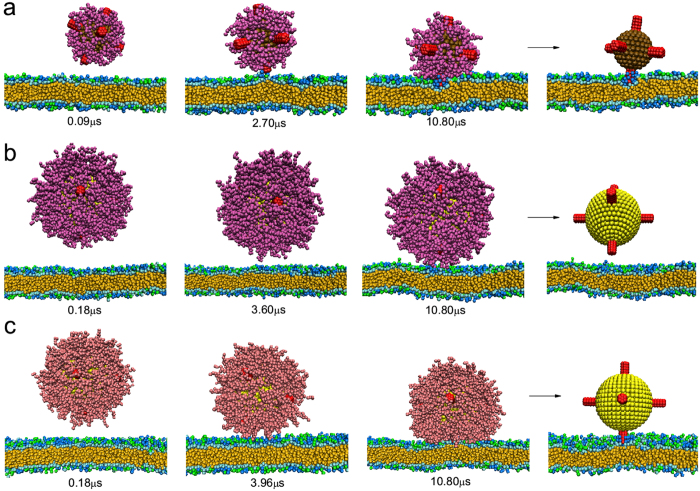
Time sequence of the snapshots of interactions between hydrophobic or charged nanoparticle with different surface coatings and cell membranes in the presence of antibody. (**a**) Hydrophobic nanoparticle decorated with hydrophilic polymers (N = 8, *σ* = 0.8/*nm*^2^) ; (**b**) positively charged nanoparticle decorated with hydrophilic polymers (N = 16, *σ* = 0.8/*nm*^2^); (**c**) positively charged nanoparticle decorated with ionized zwitterionic polymers under low pH (N = 16, *σ* = 0.8/*nm*^2^). The snapshots in the last column illustrate the final equilibrium of each simulation system (coating polymers are not shown for clarity).

As shown in Fig. 6a, in the presence of antibody, the hydrophobic nanoparticle coated with hydrophilic polymers (N = 8, *σ* = 0.8/*nm*^2^) can still interact with cell membrane. Since the interaction between antibody and receptor on cell membrane is specific (which is believed to be much larger than the electrostatic interaction^55^), the adsorption under this situation is very strong, which is beneficial for the subsequent active endocytosis. However, since the specific interaction is usually short-ranged^55^, if the length of coating polymers is very long, these polymers may also affect the cellular uptake. As illustrated in Fig. 6b, though the positively charged nanoparticle coated with hydrophilic polymers (N = 16, *σ* = 0.8/*nm*^2^) can approach the membrane due to electrostatic interaction, the antibody is not able to directly interact with receptors on the membrane since its length is within the polymer layers. Nevertheless, if the nanoparticle is coated with pH-responsive zwitterionic polymers under low pH, it may become positively charged^53^. As discussed above, its electrostatic interaction (with cell membrane) is much larger, which can enhance the attachment. More importantly, this will make the zwitterionic polymer layer deformed and in turn shorten the polymer effective length (Fig. 6c). As a result, the receptors on the membrane may be within the range of the antibody, and the specific interaction between them can induce the obvious decrease of total energy (see Table 1). In a word, our results show that this type of nanoparticle can exhibit novelly responsive behavior during the cellular delivery, namely, the antibody is protected by the coating polymers in the normal tissues or blood, and the antibody will only work until it begins to interact with targeted cells under low pH environments, which can not only prevent the antibody from degradation by enzyme but also decrease the uptake by normal cells.

**Figure 7:**
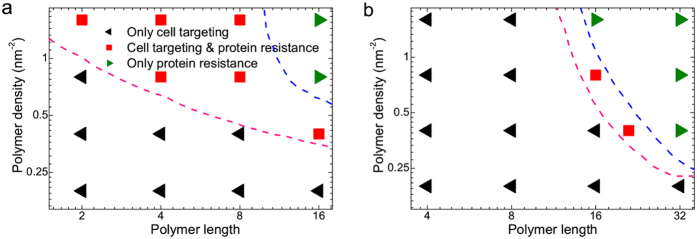
Phase diagrams describing the ability of zwitterionic polymer (ZP)-coated nanoparticles for protein resistance and active targeting on the polymer length-polymer density plane. (**a**) Hydrophobic nanoparticle with the size of 6 nm; (**b**) positively charged nanoparticle with the size of 10 nm. The region above the pink dashed line corresponds to that the nanoparticles can resist the serum proteins; while the region below the blue dashed line corresponds to that the nanoparticles can actively target the cells.

On the basis of above discussions, we can conclude that nanoparticles coated by longer polymers with larger surface density can resist the protein adsorption; while nanoparticles coated by shorter polymers with smaller surface density can still have the ability of active targeting. Thus, when decorating nanoparticles with polymers during the synthesis, it is of great importance to keep the balance between the protein resistance and active targeting. In order to provide useful design guidelines for real applications, here we give the phase diagram on the polymer length–polymer density plane (see Fig. 7). More importantly, it is found that there exists such region (corresponding to the optimal design strategy of surface decoration) in the phase diagram, where the protein resistance and cell targeting can be simultaneously achieved. Finally, notice that one polymer bead in DPD simulations usually represents one repeated monomer in real stealth polymer (e.g., PEG)^30^,^34^, thus the phase diagram here can semi-quantitatively/quantitatively provide design guideline for real applications.

In summary, we have investigated the effect of surface decoration on the cellular delivery of nanoparticles in the presence of serum protein by using DPD simulations. Our results show that hydrophilic and zwitterionic polymers can both resist the adsorption of protein onto nanoparticle surface due to their hydrophilicity, but their resistance ability is a bit different, especially in the case of positively charged nanoparticles. Apart from the polymer type, polymer surface density and length are also important factors in the protein resistance, and it is found that the surface density is more important for hydrophobic nanoparticles, and the polymer length is more important for positively charged nanoparticles. Besides, we also find that the decoration of non-specific polymers onto the nanoparticle surface may not only block the insertion of hydrophobic nanoparticles but also weaken the adsorption of positively charged nanoparticles onto the membrane, thus it will decrease the translocation efficiency of nanoparticles through membranes. By decorating antibody on the nanoparticle surface, we further make a deep discussion on the design strategy of surface decoration and also provide an optimal choice to well keep the balance between protein resistance and cell targeting from computational point of view. In general, the present study reveals the physical mechanism of polymer-coated nanoparticles interacting with proteins and cell membranes in the cellular delivery, and may be of great importance in engineering new types of nanomaterials for biomedical applications.

## Methods

The dissipative particle dynamics is a coarse-grained simulation technique with hydrodynamic interaction^30^. The dynamics of the elementary units which are so-called DPD beads, is governed by Newton’s equation of motion. Typically, there are three types of pairwise forces acting on bead *i* by bead *j* in the DPD: the conservative force, dissipative force, and random force. The conservative force 
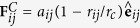
 is used to model the repulsive interaction of beads *i* and *j*, where *r*_*ij*_ = |**r**_*ij*_| is the distance between beads *i* and *j*, 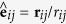 is the unit vector, *r*_*c*_ is the cutoff radius of the force, and *a*_*ij*_ represents the maximum repulsion interaction of beads *i* and *j*. For any two beads of the same type, we take the repulsive parameter *a*_*ii*_ = 25 *k*_*B*_*T*/*r*_*C*_^30^, and for any two beads of different types, the interaction parameter *a*_*ij*_ can be calculated based on the Flory-Huggins interaction parameters *χ*_*ij*_^30^: *a*_*ij*_ = *a*_*ii*_ + 3.497*χ*_*ij*_, where *χ*_*ij*_ is determined by the solubility parameter difference of the beads. Besides, if the beads do not represent any specific type, *a*_*ij*_ is set as 25 *k*_*B*_*T*/*r*_*C*_ if the two beads are both hydrophilic (notice that the charged bead is hydrophilic) or both hydrophobic; *a*_*ij*_ is set as 100 *k*_*B*_*T*/*r*_*C*_ if one is hydrophilic and the other one is hydrophobic^56^,^57^. The dissipative force and random force serve as thermostat^30^.

In the present work, the long-ranged coulomb force is also included to take into account the electrostatic interactions between charged beads. Since the soft potential in the DPD allows for the overlap between DPD beads, this can lead to the formation of artificial ion pairs and cause the divergence of the electrostatic potential when charged DPD beads are modeled. To avoid this problem, Groot chose to spread out the charges using the distribution^31^: 
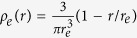
 with *r* < *r*_*e*_, where *r*_*e*_ is the electrostatic smearing radius, and is typically set as 1.6 *r*_*c*_. Besides, the soft LJ potential is used to mimic the receptor-ligand interaction^32^: 
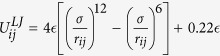
, where *r*_*ij*_ ≤ *r*_*c*_, *σ* = 0.624*r*_*c*_, and 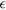 represents the strength of the receptor-ligand interaction, and is set as 5.0 *k*_*B*_*T* in the simulations. In order to guarantee the proper running of the DPD technology, the repulsive force is set to be 25 *k*_*B*_*T*/*r*_*c*_ if it is larger than 25 *k*_*B*_*T*/*r*_*c*_. Furthermore, we use a harmonic bond between the neighboring beads in a single molecule *U*_*s*_ = *k*_*s*_(1 − *r*_*i*,*i*+1_/*l*_0_)^2^ (*k*_*s*_ = 64, *l*_0_ = 0.5 *r*_*c*_) to ensure the integrality of lipids, polymers and HSA protein, where *k*_*s*_ is the spring constant and *l*_0_ is the equilibrium bond length. A weaker bond is inserted (*k*_*s*_ = 10, *l*_0_ = 0.5 *r*_*c*_) between the first hydrophobic beads on two tails of the lipid to keep the tails oriented in the same direction^24^,^56^. We also use a three-body bond angle potential *U*_*a*_ = *k*_*a*_(1 − *cos*(*ϕ* − *ϕ*_0_)) to depict the rigidity of lipid tails (*k*_*a*_ = 10, *ϕ*_0_ = 180°) and HSA protein (*k*_*a*_ = 2, *ϕ*_0_ = 180°), where *ϕ* is the angle formed by three adjacent beads in the same tail and *ϕ*_0_ is the equilibrium value of the angle.

In our simulations, the velocity-Verlet integration algorithm is used to update the coordination of each bead, where the integration time step Δ*t* is 0.015 *τ*. For the simplicity, the cutoff radius *r*_*c*_, bead mass *m*, energy *k*_*B*_*T* are chosen as the simulation units. The initial size of the simulation box is 75 *r*_*c*_ × 75 *r*_*c*_ × 40 *r*_*c*_ with the number density of 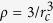. The area (*A*_0_) per lipid is about 1.28 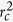 when the membrane is under zero tension at the beginning of the simulations. During the simulations, to keep the membrane surface under zero tension, the box shape changes with the area (*A*_*b*_) per lipid on the boundary, i.e., if *A*_*b*_ > *A*_0_, the box will be compressed in X-Y plane until *A*_*b*_ = *A*_0_; while if *A*_*b*_ < *A*_0_, the box will be stretched in X-Y plane until *A*_*b*_ = *A*_0_. Meanwhile, the box length in membrane-normal direction will make corresponding change to keep the box volume fixed^39^,^40^. The above operation is performed every one thousand time steps. All simulations are performed in the NVT ensembles, and the periodic boundary conditions are adopted in three directions. The DPD units can be converted into SI units by mapping the membrane thickness and the lipid diffusion coefficient^32^: *r*_*c*_ = 1.0 *nm* and *τ* = 2.4 *ns*. All simulations in this work are carried out by using the modified soft package Lammps (1 Feb 2014)^58^.

## Additional Information

**How to cite this article**: Ding, H.-m. and Ma, Y.-q. Design strategy of surface decoration for efficient delivery of nanoparticles by computer simulation. *Sci. Rep.* **6**, 26783; doi: 10.1038/srep26783 (2016).

## Supplementary Material

Supplementary Information

## Figures and Tables

**Table 1:** Summary for interaction of different types of nanoparticles with cell membranes.

Nanoparticle type	Polymer type	Antibody	*d*	Δ*E*
Hydrophobic-6 nm	None	No	0.2 ± 0.3 *nm*	219.5 ± 23.5 *kBT*
Hydrophobic-6 nm	HP (N = 8, *σ* = 0.8/*nm*^2^)	No	16.0 ± 1.5 *nm*	0 *kBT*
Hydrophobic-6 nm	HP (N = 8, *σ* = 0.8/*nm*^2^)	Yes	7.3 ± 0.2 *nm*	−265.3 ± 23.1 *kBT*
Hydrophobic-6 nm	ZP (N = 8, *σ* = 0.8/*nm*^2^)	No	12.8 ± 0.6 *nm*	−2.6 ± 0.5 *kBT*
Hydrophobic-6 nm	ZP (N = 8, *σ* = 0.8/*nm*^2^)	Yes	7.2 ± 0.2 *nm*	−276.0 ± 26.4 *kBT*
N-charged-10 nm	None	No	18.4 ± 2.0 *nm*	−8.6 ± 1.8 *kBT*
N-charged-10 nm	HP (N = 16, *σ* = 0.8/*nm*^2^)	No	17.1 ± 2.2 *nm*	−6.8 ± 1.1 *kBT*
N-charged-10 nm	ZP (N = 16, *σ* = 0.8/*nm*^2^)	No	19.2 ± 1.7 *nm*	−10.6 ± 2.0 *kBT*
P-charged-10 nm	None	No	7.4 ± 0.4 *nm*	−150.6 ± 12.3 *kBT*
P-charged-10 nm	HP (N = 16, *σ* = 0.8/*nm*^2^)	No	12.7 ± 0.2 *nm*	−18.6 ± 1.9 *kBT*
P-charged-10 nm	HP (N = 16, *σ* = 0.8/*nm*^2^)	Yes	12.4 ± 0.4 *nm*	−21.4 ± 2.8 *kBT*
P-charged-10 nm	ZP (N = 16, *σ* = 0.8/*nm*^2^)	No	12.5 ± 0.3 *nm*	−23.6 ± 2.4 *kBT*
P-charged-10 nm	ZP (N = 16, *σ* = 0.8/*nm*^2^)	Yes	12.2 ± 0.2 *nm*	−30.1 ± 3.2 *kBT*
P-charged-10 nm	ZP-pH (N = 16, *σ* = 0.8/*nm*^2^)	No	11.0 ± 0.2 *nm*	−110.4 ± 10.8 *kBT*
P-charged-10 nm	ZP-pH (N = 16, *σ* = 0.8/*nm*^2^)	Yes	10.1 ± 0.2 *nm*	−320.5 ± 40.2 *k*_*B*_*T*

Hydrophobic-6 nm represents the hydrophobic nanoparticle with the size of 6 nm; N-charged-10 nm represents the negatively charged nanoparticle with the size of 10 nm; P-charged-10 nm represents the positively charged nanoparticle with the size of 10 nm; *d* is defined as the distance between the center of mass of nanoparticle core to the middle plane of cell membranes; Δ*E* is defined as the difference of total energy of nanoparticle (including polymers and antibody) and cell membranes between the initial and final state. ±Sign indicates standard error of mean (n = 5).
