# Supplementary material for: Design strategy of surface decoration for efficient delivery of nanoparticles by computer simulation
Source: Sci Rep. 2016 May 26;6:26783. doi: 10.1038/srep26783 (PMC4880933; doi:10.1038/srep26783)
Supplement: Supplementary Information [file srep26783-s1.pdf]

# Supplementary Information for:
# Design strategy of surface decoration for efficient delivery of nanoparticles by computer simulation

Hong-ming Ding[1] & Yu-qiang Ma[1,2,*]

[1] *Center for Soft Condensed Matter Physics and Interdisciplinary Research, Soochow University, Suzhou 215006, China*

[2] *National Laboratory of Solid State Microstructures and Department of Physics, Collaborative Innovation Center of Advanced Microstructures, Nanjing University, Nanjing 210093, China*

* *E-mail address: myqiang@nju.edu.cn*

## I. Supplementary Figures

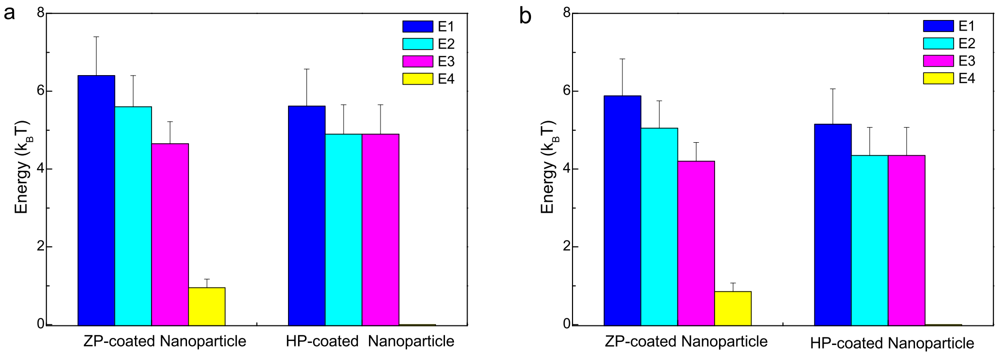


Figure S1: The averaged interaction energy between coating polymers and HSA protein after the adsorption of proteins onto different nanoparticles, where E1 represents the interaction energy between coating polymers and HSA protein; E2 stands for the interaction energy between the ending two beads of coating polymers and HSA protein (E3 is the hydrophobic interaction and E4 is the electrostatic interaction). (a) The polymer density is $0.8/nm^2$ and the polymer length is 4; (b) the polymer density is $0.8/nm^2$ and the polymer length is 8.

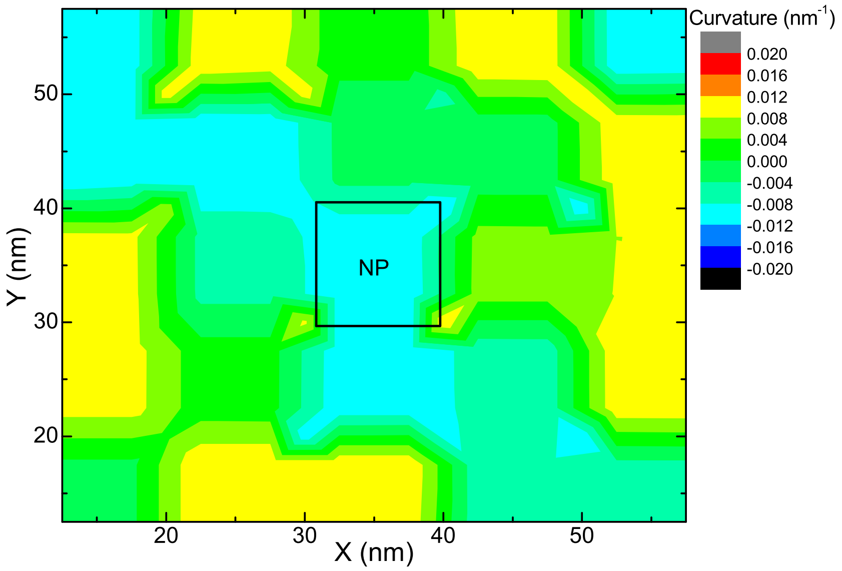


Figure S2: The estimated curvature of cell membrane on the X-Y plane. The inset rectangular box indicates the location of zwitterionic polymer-coated nanoparticle on the cell membrane, and the mean curvature of this region is about $-0.01nm^{-1}$.

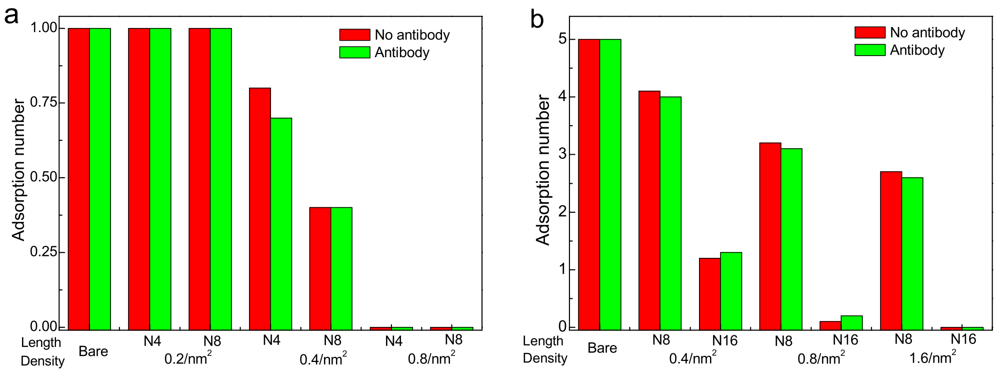


Figure S3: Comparison of interaction of HSA protein with nanoparticles in the absence and presence of antibody molecules. (a) The adsorption number of HSA protein on hydrophobic nanoparticle (6 nm) surface as functions of the property (i.e., polymer length and density) of coating zwitterionic polymers. (b) The adsorption number of HSA protein on positively charged nanoparticle (10 nm) surface as functions of the property (i.e., polymer length and density) of coating zwitterionic polymers. Ten independent simulations are averaged.
